# Placebo and Nocebo Effects: The Advantage of Measuring Expectations and Psychological Factors

**DOI:** 10.3389/fpsyg.2017.00308

**Published:** 2017-03-06

**Authors:** Nicole Corsi, Luana Colloca

**Affiliations:** ^1^Department of Pain Translational Symptom Science, School of Nursing, University of MarylandBaltimore, MD, USA; ^2^Department of Neurosciences, Biomedicine and Movement Sciences, University of VeronaVerona, Italy; ^3^Department of Anesthesiology/Psychiatry, School of Medicine, University of MarylandBaltimore, MD, USA; ^4^Center to Advance Chronic Pain Research, University of MarylandBaltimore, MD, USA

**Keywords:** acute pain, anxiety, conditioning, expectation, fear, neuroticism, suggestibility

## Abstract

Several studies have explored the predictability of placebo and nocebo individual responses by investigating personality factors and expectations of pain decreases and increases. Psychological factors such as optimism, suggestibility, empathy and neuroticism have been linked to placebo effects, while pessimism, anxiety and catastrophizing have been associated to nocebo effects. We aimed to investigate the interplay between psychological factors, expectations of low and high pain and placebo hypoalgesia and nocebo hyperalgesia. We studied 46 healthy participants using a well-validated conditioning paradigm with contact heat thermal stimulations. Visual cues were presented to alert participants about the level of intensity of an upcoming thermal pain. We delivered high, medium and low levels of pain associated with red, yellow and green cues, respectively, during the conditioning phase. During the testing phase, the level of painful stimulations was surreptitiously set at the medium control level with all the three cues to measure placebo and nocebo effects. We found both robust placebo hypolagesic and nocebo hyperalgesic responses that were highly correlated with expectancy of low and high pain. Simple linear regression analyses showed that placebo responses were negatively correlated with anxiety severity and different aspects of fear of pain (e.g., medical pain, severe pain). Nocebo responses were positively correlated with anxiety sensitivity and physiological suggestibility with a trend toward catastrophizing. Step-wise regression analyses indicated that an aggregate score of motivation (value/utility and pressure/tense subscales) and suggestibility (physiological reactivity and persuadability subscales), accounted for the 51% of the variance in the placebo responsiveness. When considered together, anxiety severity, NEO openness-extraversion and depression accounted for the 49.1% of the variance of the nocebo responses. Psychological factors *per se* did not influence expectations. In fact, mediation analyses including expectations, personality factors and placebo and nocebo responses, revealed that expectations were not influenced by personality factors. These findings highlight the potential advantage of considering batteries of personality factors and measurements of expectation in predicting placebo and nocebo effects related to experimental acute pain.

## Introduction

Personality factors can influence placebo and nocebo effects (Colloca and Grillon, 2014; Colagiuri et al., 2015). Factors such as dispositional optimism (Geers et al., 2005, 2007, 2010; Nes and Segerstrom, 2006; Morton et al., 2009), hypnotic suggestibility (De Pascalis et al., 2002), somatic focus (Geers et al., 2006; Johnston et al., 2012), empathy (Colloca and Benedetti, 2009; Hunter et al., 2014; Rütgen et al., 2015a,b), neuroticism (Peciña et al., 2013), altruism (Peciña et al., 2013), social desirability (Gelfland et al., 1965), dopamine-related traits (Schweinhardt et al., 2009), fear of pain (Flaten et al., 2006; Zubieta et al., 2006; Lyby et al., 2010), locus of ego-resilience (Peciña et al., 2013), anxiety (Staats et al., 2001; Ober et al., 2012), pessimism (Geers et al., 2005; Corsi et al., 2016), pain catastrophizing (Vogtle et al., 2013), harm avoidance, and persistence (Corsi et al., 2016) have been linked to placebo and nocebo effects.

In particular, optimism, the active behavioral and mental coping ability of individuals to face adversity, has been liked to proneness to show higher placebo analgesic effects (Geers et al., 2005, 2007, 2010). Attention toward the body, referred as somatic focus, is related to larger placebo analgesic effects and higher positive expectations (Geers et al., 2006). Empathic resonance and concern for others have been linked to placebo analgesia as well (Colloca and Benedetti, 2009; Hunter et al., 2014; Rütgen et al., 2015a,b). Hypnotic susceptibility and responsiveness to verbal suggestions influence placebo analgesia (Huber et al., 2013). Other factors such as Neuroticism-Extraversion-Openness to experience (NEO), NEO Altruism, NEO Straightforwardness, NEO Angry Hostility and Ego-Resiliency, have been coupled with a 25% variance in behavioral placebo responses to pain and 27% of the μ-opioid system activation in the nucleus accumbens (Peciña et al., 2013).

Conversely, anxiety (Staats et al., 2001), harm avoidance and persistence (Corsi et al., 2016) and pain catastrophizing (Swider and Babel, 2013; Vogtle et al., 2013) have been associated with nocebo effects. Anxiety and harm avoidance correlate positively with nocebo effects, while optimism and persistence correlate negatively with nocebo effects in the context of the motor system (Corsi et al., 2016). In the present study, our aim was to investigate how distinct positive and negative personality factors estimate the likelihood of placebo and nocebo effects. Moreover, we aimed to establish the relationship among trial-by-trial expectations of pain reduction and increase, and placebo/nocebo effects, and personality. We hypothesized that using aggregated personality factors and expectations would allow us to better estimate placebo and nocebo responses in a laboratory setting using a well-established conditioning model (Colloca et al., 2010).

## Materials and methods

### Study participants

We recruited 50 participants from Baltimore, MD, USA to enroll a total of 46 healthy participants (24 women; 27.41 ± 1.07 years; see Table 1). Four participants were excluded: two of them did not meet the inclusion criteria and two were unable to discriminate distinct levels of heat thermal stimulation that are used for the acquisition phase of the conditioning paradigm. Upon arrival, participants signed a consent form to study pain modulation. Participants with cardiovascular and neurological diseases, family or personal history of psychiatric conditions, personal history of drug abuse, acute or chronic pain, color blindness, impaired hearing, pregnancy and current use of painkillers and any other medication, were excluded from participating in this study. On the day of the experiment, a toxicology drug test was also performed to exclude any recent use of marijuana, cocaine, opiates such as hydrocodone, oxycodone and hydromorphone, amphetamine, methamphetamine, ecstasy/MDMA and phencyclidine. Participants who reported use of tobacco or nicotine over the last year were also excluded.

**Table 1:** **Characteristics of study participants**.

**Characteristics of Participants**	
Sex	24 females
	22 males
Age (years)	27.41 ± 1.07
Body Mass Index (BMI)	26.00 ± 0.71
Systolic blood pressure values (mmHg)	120.19 ± 2.00
Diastolic blood pressure (mmHg)	75.15 ± 1.27
Heart rate (beats per minute)	66.36 ± 1.45
Levels of pain (°C)	Low 41.51 ± 0.36
	Medium 44.55 ± 0.36
	High 47.52 ± 0.36

*All values are expressed as mean ± SE*.

This study was carried out in accordance with the recommendations of the UMB Institutional Review Board with written informed consent from all subjects.

All subjects gave written informed consent in accordance with the Declaration of Helsinki. The protocol was approved by the UMB Ethics Committee (Prot # HP00065783). Due to the use of deception, a debriefing written form was given to each participant at the end of the study participation offering to withdraw the data from the study. None of them opted to do so. Participants were compensated for their participation ($90).

### Pain assessment

A well-validated paradigm that has been previously described (Colloca et al., 2010) was used to explore placebo and nocebo responses to a contact heat thermal painful stimulation.

Individual pain sensitivity and tolerance were measured in each participant using the ATS Medoc Pathway system (Medoc Advanced Medical System, Rimat Yishai, Israel). A 3 × 3 cm thermode was placed on the dominant forearm as confirmed by the Edinburgh Handedness Inventory. The baseline temperature delivered by the Medoc equipment was 32°C. Ascending series of stimulations starting from warm sensation to maximum tolerable pain were delivered, while the participant was asked to stop the machine as soon as she felt a warm sensation, low, medium and high pain. Each level was assessed four times and averaged to determine the intensities of stimulations to be used during the acquisition and testing phases of the conditioning paradigm. We defined then the painful stimulations by subtracting 3 and 6°C starting from the highest reported level of tolerable pain (e.g., 49 and 43°C) so that the levels of stimulation were standardized among participants. The intensities of stimulation were also rated to ensure correspondence to individual experience of low, medium and high pain.

### Placebo and nocebo manipulation

Three visual cues (red, yellow, and green) were displayed on a computer placed one meter apart from a chair in a quiet lab. Participants were told that the green, yellow and red lights would anticipate the delivery of a low, medium and high level of pain, respectively.

During the acquisition phase of the classical conditioning paradigm, 18 painful stimulations were delivered at the three levels of pain corresponding to an individual low, medium, and high level of pain in association to six red, six yellow, and six green cues, respectively. Afterwards, during the testing phase, 9 stimulations were paired with the three color cues but the intensity was set at same medium control level in accordance with a previously described paradigm (Colloca et al., 2010). The sequence of the cue presentation was counterbalanced across participants using four distinct sequences. This change in the pain levels allowed us to explore how first-hand experience of low and high pain during the acquisition phase results in placebo and nocebo responses during the testing phase. Participants rated the experienced pain immediately after the painful stimulation using the VAS scale (from 0 = no pain to 100 = maximum tolerable pain). Pain reports were collected using Celeritas Fiber Optic Response System (Psychology Software Tools, Inc., Sharpsburg, PA, USA).

Moreover, trial-by-trial expectations were measured. The terms “expectation” and “expectancy” have been often used in an interchangeable way. Herein, we adopted the term “expectation” to refer to verbalized and measurable constructs as compared to “expectancies” defining psychophysical predictions that can be present without full awareness (i.e., implicit expectancies) (Kube et al., 2017).

Participants were asked to rate their expectations of the upcoming stimulation immediately before the delivery of the thermal stimulation using a VAS anchored from 0 = no pain to 100 = maximum tolerable pain.

During each trial, the visual cue was presented for 4 s. Immediately after the presentation of the cue, participants were asked to rate their expectation (5 s) about the upcoming stimulus. The thermal stimulation lasted for 10 s. Then participants were asked to rate their perceived pain (5 s) and an inter-trial interval followed with a variable timing (8–10 s). The procedure and the delivery of painful stimulations were controlled by scripts pre-programmed in Eprime (Psychology Software Tools, Inc., Sharpsburg, PA, USA; version 2.0). To prevent habituation, the presentation of visual cues during both phases was counterbalanced using four preprogramed sequences.

### Psychological questionnaires

Participants completed a comprehensive battery of psychological questionnaires, which were chosen to cover distinct psychological factors that we hypothesized to be linked to placebo and nocebo effects. In particular, for the placebo-related factors, we included optimism, reward, suggestibility, empathy and sensation-seeking and motivation. We used the following questionnaires: (1) Life-Orientation Test-Revisited, Lot-R (Scheier et al., 1994) to assess generalized optimism vs. pessimism; (2) Behavioral Inhibition and Behavioral Activation Scale, BIS/BAS (Carver and White, 1994) to investigate dispositional sensitivity to the behavioral inhibition system (BIS) and the behavioral activation system (BAS); (3) Multidimensional Iowa Suggestibility Scale, MISS (Kotov et al., 2004) to investigate the main components of suggestibility; (4) Interpersonal Reactivity Index, IRI (Davis, 1980) to measure the participant's dispositional empathy in different situations; (5) Sensation Seeking (SS) (Zuckerman, 1994) to measure the necessity to find and experience new situations; (6) Tri-dimensional Personality Questionnaire, TPQ (Cloninger et al., 1991) to assess novelty seeking (NS), harm avoidance (HA), and reward dependence (RD); (7) and the Intrinsic Motivation Inventory (IMI) (Markland and Hardy, 1997) to assess participants' experience during the experimental procedure that was just performed.

For the nocebo-related psychological factors included measurements of various aspects of anxiety (e.g., state, severity, and sensitivity), catastrophizing, neuroticism, fear of pain, depression and feelings of worry. The following inventories were used: (1) State and Trait Anxiety Inventory, STAI (Spielberger, 1983) to investigate anxiety either in a precise moment (STAI-Y1) or as a general tendency (STAI-Y2); (2) Anxiety Sensitivity Index, ASI (Reiss et al., 1986) to assessed beliefs of sensations that could have harmful consequences; (3) Beck Anxiety Inventory, BAI (Beck et al., 1988) to measure experience of anxiety symptoms during the previous 2 weeks; (4) Beck Depression Inventory, BDI (Beck et al., 1961) to include items relating to depression, cognitions, as well as physical symptoms; (5) Mood and Anxiety Symptom Questionnaire, MASQ (Haigh et al., 2011) to assess depressive symptoms and anxiety symptoms; (6) Pain Catastrophizing Scale, PCS (Sullivan et al., 1995) to assess catastrophizing impacts on pain experience; (7) Neuroticism—Extroversion—Openness Inventory (NEO)—Five Factory Inventory (FFI) (Costa and McCrae, 1985, 1992) to investigate Neuroticism, Extraversion, Openness to Experience, Agreeableness, and Conscientiousness; (8) Fear of Pain Questionnaire, FOP (Osman et al., 2002) to measure fear levels to different types of physical pain; (9) Penn State Worry Questionnaire, PSWQ (Meyer et al., 1990) to measure the trait of worry in different situations.

We also administered the Positive and Negative Affective Schedule, PANAS (Crawford and Henry, 2004), that investigates the relationships between positive and negative affect with personality states and emotions.

### Statistical analysis

VAS pain and VAS expectations ratings were compared using repeated measure ANOVA. We tested for the main effect of the factor condition (red, yellow, and green) and time (trials) set both as within-subjects factors. *F*-tests were followed by the Bonferroni *post-hoc* tests for multiple comparisons. We also tested for sex influences on placebo and nocebo effects using sex as a between factor. Partial eta squared (η^2^) effect sizes are reported for all the comparisons.

VAS pain and expectation scores from the testing phase were further averaged across trials to calculate the difference between yellow-green and yellow-red pain scores to be correlated with placebo and nocebo effects, respectively.

The above psychological questionnaire scores were used in both simple correlation and multivariate analyses. We analyzed psychological questionnaire scores using both Spearman correlation and stepwise multiple regression model analyses in which the questionnaires were modeled to predict placebo and nocebo responses. Mediation analyses were also calculated with expectation as mediator (M), placebo (or nocebo) responses as dependent variable (Y), and personality factors as independent variable (X). For testing indirect effects, a bootstrapping method based on resampling of 1,000 times was used in accordance with Preacher and Hayes methods (Preacher and Hayes, 2004; Hayes and Preacher, 2010). All the analyses were carried out using the SPSS software package (SSPS Inc, Chicago, Illinois, USA, vers.21). To minimize alpha errors, the level of significance was set at *p* ≤ 0.005.

## Results

We performed separate analyses for the VAS pain and expectation reports related to the acquisition and testing phases of the conditioning paradigm.

### Conditioning: acquisition phase

**Figure 1:**
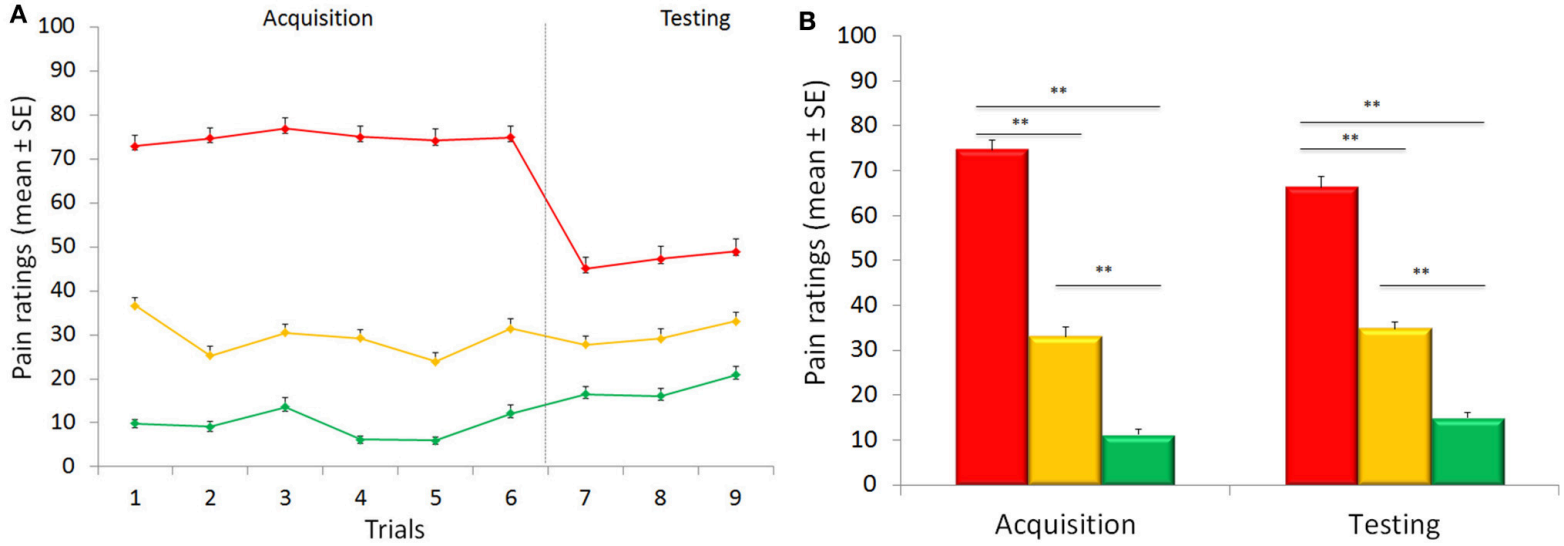
**Time course of placebo and nocebo responses (A)**. Representation trial-by-trial of the average of pain ratings for control (yellow), placebo (green) and nocebo (red) responses during the acquisition (trials 1–6) and the testing (trials 7–9) phases. Participants learned to distinguish the low, medium and high levels of painful stimuli over the acquisition phase. During the testing phase, there was a significant placebo and nocebo effect indicating no extinction over the entire experimental session. Graphical representation of the pain score for the red, green, and yellow associated stimuli **(B)**. The red associated stimuli were perceived as higher than the yellow control stimuli and green were rated as lower than the yellow stimuli during the testing phases when the stimulation was surreptitiously set at a medium level for the three colors indicating both robust placebo and nocebo effects. Data are expressed as mean ± sem. ^**^p < 0.001.

We analyzed the VAS pain reports during the acquisition phase, and found that participants distinguished the low, medium and high levels of painful stimuli [main effect of condition: *F*_(2, 88)_ = 503.970, *p* < 0.001, η^2^ = 0.920]. The average pain score for red-associated stimuli was 74.73 ± 2.36 using an average intensity of pain equal to 47.52°C, the average pain score for yellow was 29.55 ± 1.54 using an average pain equal to 44.55°C and the average pain score for green was 9.37 ± 0.96 when an average pain equal to 41.51°C out of 50°C was delivered. The factor time was significant [*F*_(5, 220)_ = 7.359, *p* < 0.001, η^2^ = 0.143]. The condition × time interaction was significant [*F*_(10, 440)_ = 5.324, *p* < 0.001, η^2^ = 0.108] (Figure 1A) showing a quadratic trajectory [*F*_(1, 44)_ = 10.308, *p* < 0.002, η^2^ = 0.190].

**Figure 2:**
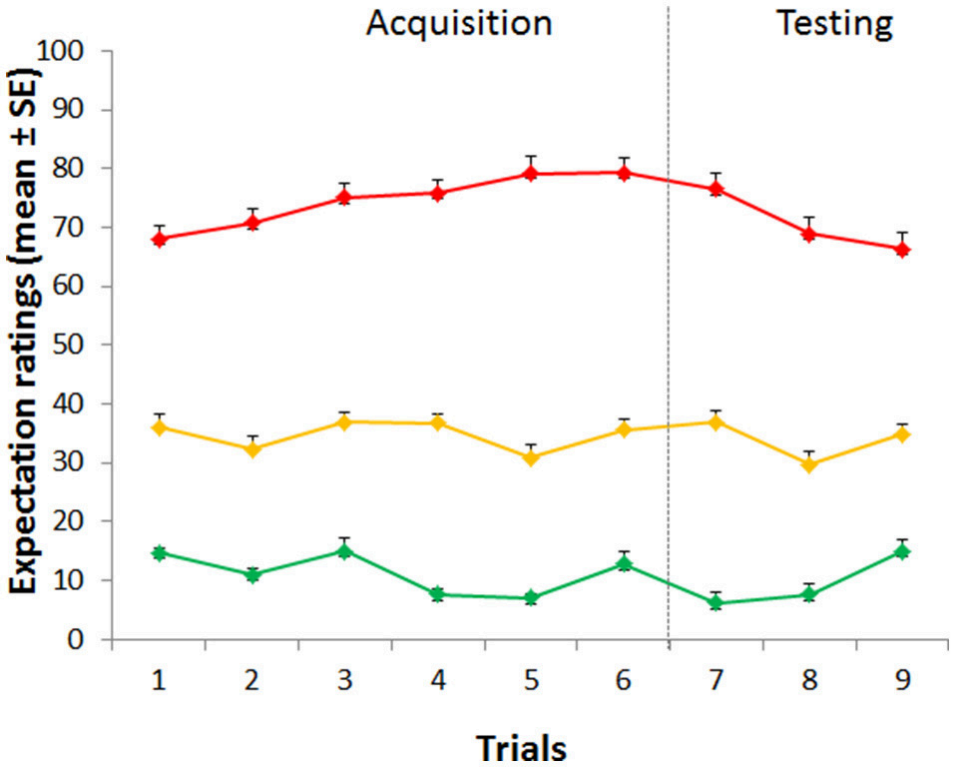
**Time course of expectation ratings**. Expectations during the acquisition phase differed across the three conditions. During the testing phase, expectations for high, medium and low pain continued to be staidly different across the three conditions.

VAS expectation scores (75.63 ± 2.09, 34.74 ± 1.61, and 11.30 ± 0.98, respectively) during the acquisition phase differed across the three conditions [*F*_(2, 88)_ = 515.152, *p* < 0.001; η^2^ = 0.921], with significant time [*F*_(5, 220)_ = 3.392, *p* = 0.006; η^2^ = 0.072] and condition × time interaction [*F*_(10, 440)_ = 7.542, *p* < 0.001; η^2^ = 0.146] effects (Figure 2).

### Conditioning: testing phase

**Figure 3:**
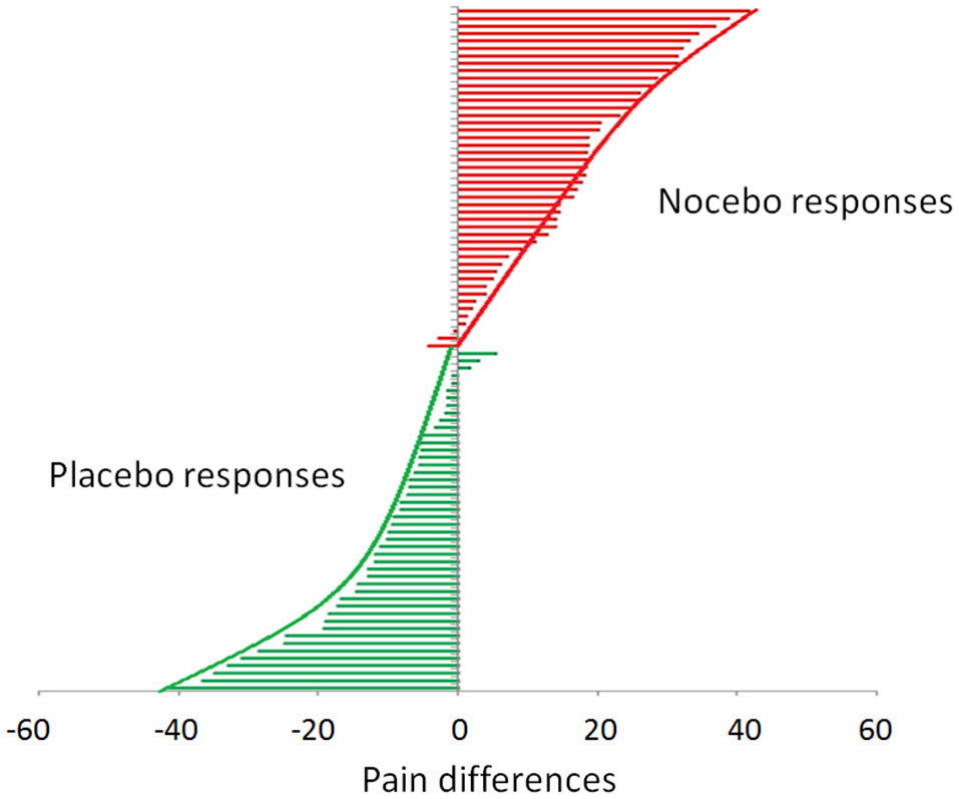
**Distribution of placebo and nocebo effects**. Each bar represents a single study participant. The green bars represent the magnitude of the placebo effect (yellow-green VAS scores). The red bars represent the magnitude of the nocebo effect (yellow-red-VAS score). It is worth noting that the individual placebo and nocebo responses range from no responses at all to medium to large effect.

During the testing phase, when the level of pain was set at the same control (yellow) intensity for the three cues, VAS pain reports revealed a significant effect of condition [*F*_(2, 88)_ = 96.04, *p* < 0.001; η^2^ = 0.686], time [*F*_(2, 88)_ = 7.553, *p* = 0.001; η^2^ = 0.147] with a non-significant condition × time interaction [*F*_(4, 176)_ = 0.378, *p* = 0.824; η^2^ = 0.009] indicating no extinction over the entire experimental session (Figure 1A). *Post-hoc* Bonferroni tests indicated that the red stimuli (average VAS: 46.98 ± 2.46) were perceived as higher than the yellow control stimuli (average VAS: 29.96 ± 1.78) (*p* < 0.001) and green (average VAS: 17.86 ± 1.70) were rated as lower than the yellow stimuli (*p* < 0.001) indicating both robust placebo and nocebo effects (Figure 1B). The distribution and magnitude of placebo and nocebo responses ranged from no effects to large changes in pain modulation (Figure 3).

Placebo effects were significantly correlated with the hypoalgesic effect experienced during the acquisition phase (Placebo: *r* = 0.388, *p* = 0.008) but nocebo hyperalgesic responses appeared to be independent of the experienced high pain (*r* = 0.080, *p* = 0.598). Moreover, being prone to experience a placebo response did not imply being also prone to experience a nocebo response, as indicated by the absence of significant correlation between individual placebo and the nocebo responses (*r* = −0.113, *p* = 0.454).

**Figure 4:**
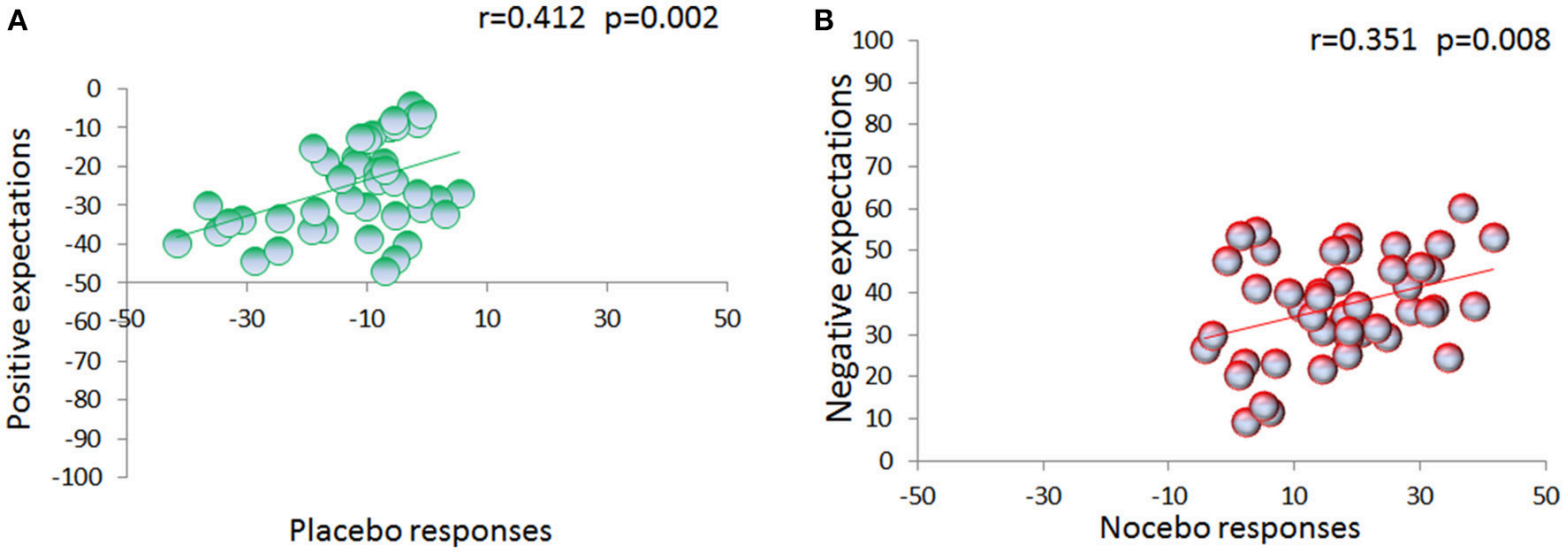
**Relation between expectations and placebo/nocebo effects**. VAS expectation scores were collected on a trial-by-trial basis during the testing phase. Expectation of low pain positively correlates with placebo effects **(A)**. Similarly, expectation of upcoming high painful stimulation positively correlates with nocebo effects **(B)**.

During the testing phase, expectations for high, medium and low pain [70.61 ± 2.45, 33.87 ± 1.81, and 9.54 ± 0.93] were different across the three conditions [*F*_(2, 88)_ = 441.355, *p* < 0.001; η^2^ = 0.909] with a main effect of time [*F*_(2, 88)_ = 8.092, *p* = 0.001; η^2^ = 0.155], and a significant interaction condition × time [*F*_(4, 176)_ = 13.156, *p* < 0.001; η^2^ = 0.230] (Figure 3), showing a linear trajectory [*F*_(1, 44)_ = 33.850, *p* < 0.001, η^2^ = 0.435]. Importantly, we found that positive expectations correlated with placebo responses (*r* = 0.412, p = 0.002, Figure 4A) and similarly negative expectations correlated with nocebo effects (*r* = 0.351, *p* = 0.008, Figure 4B).

In this cohort of participants, sex effects for placebo, nocebo and expectancies were not observed [placebo: *F*_(1, 44)_ = 0.010, *p* = 0.922; nocebo: *F*_(1, 44)_ = 0.990, *p* = 0.325; positive expectancies: *F*_(1, 44)_ = 1.860, *p* = 0.180; negative expectancies: *F*_(1, 44)_ = 0.025, *p* = 0.875].

### Personality predictors

We then explored the effects of personality factors on placebo and nocebo effects. First, we ran a series of correlations analyses and found that placebo responses were negatively correlated with severity of anxiety (BAI: *r* = −0.485, *p* = 0.001), and fear of pain (FOP, severe: *r* = −0.490, *p* = 0.001; medical fear, *r* = −0.416, *p* = 0.004; total fear *r* = −0.435, *p* = 0.003). By the contrary, nocebo responses were positively correlated with anxiety sensitivity (ASI, *r* = 0.460, *p* = 0.001), physiological suggestibility (MISS: *r* = 0.438, *p* = 0.002) with a trend for catastrophizing tendency (PCS rumination: *r* = 0.352, *p* = 0.016; PCS helplessness: *r* = 0.366, *p* = 0.012; PCS total: *r* = 0.343, *p* = 0.020) (Table 2).

**Table 2:** **Correlations between placebo, nocebo and personality factors**.

**Personality factors**	**PLACEBO EFFECT**		**NOCEBO EFFECT**	
	***R***	***p***	***r***	***p***
STAI-Y1 pre	−0.175	0.246	0.217	0.147
STAI-Y2 post	−0.004	0.978	0.177	0.241
STAY2	−0.123	0.415	0.119	0.432
ASI	−0.147	0.330	**0.460**	**0.001**
BAI	−**0.485**	**0.001**	−0.028	0.855
BDI	−0.039	0.796	0.244	0.102
PANAS total	0.096	0.527	0.175	0.245
PANAS positive	−0.218	0.145	−0.199	0.429
PANAS negative	−0.366	0.012	0.248	0.097
NEO neuroticism	−0.166	0.270	0.021	0.892
NEO extraversion	−0.349	0.018	0.186	0.217
NEO openness	−0.228	0.128	−0.264	0.076
NEO agreeableness	−0.098	0.518	0.091	0.548
NEO conscientiousness	−0.001	0.993	0.054	0.720
MASQ General depressive scale	−0.75	0.622	−0.051	0.735
MASQ anxious arousal	0.021	0.889	−0.098	0.519
MASQ general distress	−0.146	0.332	0.155	0.305
MASQ anhedonia	0.015	0.620	0.151	0.316
TPQ novelty seeking	0.015	0.922	0.045	0.769
TPQ harm avoidance	−0.164	0.277	0.099	0.511
TPQ reward dependence	−0.191	0.204	0.179	0.234
BAS drive	0.165	0.273	−0.016	0.918
BAS fun	0.000	0.999	−0.139	0.356
BAS reward	0.004	0.977	−0.32	0.382
BIS	−0.065	0.670	−0.091	0.549
BIS/BAS total	0.013	0.934	−0.144	0.341
LotR	−0.072	0.635	0.012	0.935
IMI interest/enjoyment	−0.075	0.620	−0.081	0.595
IMI perceived competence	−0.280	0.060	−0.022	0.883
IMI effort/importance	−0.142	0.346	−0.220	0.143
IMI pressure/tense	−0.017	0.910	0.295	0.047
IMI choice	−0.094	0.540	−0.197	0.194
IMI value/utility	−0.343	0.020	−0.216	0.149
IMI total	−0.339	0.021	−0.144	0.341
IRI fantasy	−0.290	0.050	−0.016	0.915
IRI empathic concern	−0.231	0.123	0.157	0.297
IRI perspective-taking	−0.189	0.208	0.093	0.538
IRI personal distress	0.190	0.207	0.195	0.193
MISS suggestibility	−0.165	0.274	0.254	0.089
MISS persuadability	−0.060	0.693	−0.016	0.918
MISS physiological suggestibility	−0.264	0.076	**0.438**	**0.002**
MISS physiological reactivity	−0.354	0.016	0.159	0.292
MISS peer conformity	−0.270	0.069	0.293	0.048
MISS mental control	−0.220	0.141	0.005	0.975
MISS unpersuadability	−0.121	0.421	0.295	0.047
MISS short suggestibility	−0.284	0.056	0.175	0.245
MISS total	−0.331	0.025	0.301	0.042
FOP severe	−**0.490**	**0.001**	−0.073	0.629
FOP medical	−**0.416**	**0.004**	−0.013	0.929
FOP total	−**0.435**	**0.003**	−0.037	0.806
PCS rumination	−0.104	0.490	0.352	0.016
PCS magnification	0.032	0.831	0.054	0.721
PCS helplessness	0.021	0.887	0.366	0.012
PCS total	−0.022	0.883	0.343	0.020
PSWQ	−0.216	0.149	0.283	0.057
SS boredom susceptibility	−0.066	0.661	0.098	0.518
SS disinhibition	−0.031	0.839	−0.027	0.861
SS experience seeking	0.078	0.605	−0.093	0.537
SS adventure seeking	−0.036	0.812	−0.025	0.869
SS total	0.014	0.924	0.005	0.976

*STAI 1-2, State and Trait Anxiety Inventory; ASI, Anxiety Sensitivity Index; BAI, Beck Anxiety Inventory; BDI, Beck Depression Inventory; PANAS, Positive and Negative Affective Schedule; NEO, Neuroticism-Extraversion-Openness Inventory; MASQ, Mood and Anxiety Symptom Questionnaire; TPQ, Tridimensional Personality Questionnaire; BISBAS, Behavioral Inhibition and Behavioral Activation Scale; LotR, Life-Orientation Test-Revisited; IMI, Intrinsic Motivation Inventory; IRI, Interpersonal Reactivity Index; MISS, Multidimensional Iowa Suggestibility Scale; FOP, Fear of Pain; PCS, Pain Catastrophizing Scale; PSWQ, Penn State Worry Questionnaire; SS, Sensation Seeking. Significant results are indicated in bold*.

Moreover, we considered the hypothesized psychological factors taken together in order to identify their relationship with the dependent variables (e.g., placebo and nocebo VAS) using stepwise multiple regression models. The significant values are reported in Tables 3, 4. Motivation (value/utility and pressure/tense subscales) and suggestibility (physiological reactivity and persuadability subscales) accounted for 51% of variance in placebo responses (Table 3). Conversely, ASI, NEO-openness-extraversion and depression taken together accounted for 49.1% of variance in nocebo responses (Table 4).

**Table 3:** **Stepwise multiple regression models for the prediction of placebo effects**.

**Dependent variable**	**Predictor Variables**	***R*****^2^**	**β**	***t***	***p***
Placebo hypoalgesia	*Model 1*	21.6			
	MISS physiol		0.464	3.438	0.001
Placebo hypoalgesia	*Model 1*	21.6			
	MISS physiol		0.464	3.438	0.001
	*Model 2*	35.4			
	MISS physiol		0.577	4.452	<0.001
	IMI value		−0.389	−2.999	0.005
Placebo hypoalgesia	*Model 1*	21.6			
	MISS physiol		0.464	3.438	0.001
	*Model 2*	35.4			
	MISS physiol		0.577	4.452	<0.001
	IMI value		−0.389	−2.999	0.005
	*Model 3*	42.7			
	MISS physiol		0.579	4.687	<0.001
	IMI value		−0.371	−2.993	0.005
	MISS persuadability		0.270	2.280	0.028
Placebo hypoalgesia	*Model 1*	21.6			
	MISS physiol		0.464	3.438	0.001
	*Model 2*	35.4			
	MISS physiol		0.577	4.452	<0.001
	IMI value		−0.389	−2.999	0.005
	*Model 3*	42.7			
	MISS physiol		0.579	4.687	<0.001
	IMI value		−0.371	−2.993	0.005
	MISS persuadability		0.270	2.280	0.028
	*Model 4*	51.0			
	MISS physiol		0.463	3.745	0.001
	IMI value		−0.335	−2.871	0.007
	MISS persuadability		0.344	3.006	0.005
	IMI pressure		0.319	2.617	0.012

*MISS, Multidimensional Iowa Suggestibility Scale (Physiological Reactivity and Persuadability subscales); IMI, Intrinsic Motivation Inventory (Value/Utility and Pressure/Tense subscales). Only significant values are shown. Excluded variables (not significant): Lot-R, Life-Orientation Test-Revisited; BIS/BAS, Behavioral Inhibition and Behavioral Activation Scale; IRI, Interpersonal Reactivity Index; SS, Sensation Seeking; TPQ, Tri-dimensional Personality Questionnaire*.

**Table 4:** **Stepwise multiple regression models for the prediction of nocebo effects**.

**Dependent variable**	**Predictor Variables**	***R*****^2^**	**β**	***t***	***p***
Nocebo hyperalgesia	*Model 1*	20.3			
	ASI		0.451	3.349	0.002
Nocebo hyperalgesia	*Model 1*	20.3			
	ASI		0.451	3.349	0.002
	*Model 2*	33.3			
	ASI		0.498	3.966	<0.001
	NEO_O		−0.364	−2.897	0.006
Nocebo hyperalgesia	*Model 1*	20.3			0.002
	ASI		0.451	3.349	
	*Model 2*	33.3			
	ASI		0.498	3.966	<0.001
	NEO openess		−0.364	−2.897	0.006
	*Model 3*	42.9			
	ASI		0.493	4.197	<0.001
	NEO openess		−0.472	−3.796	<0.001
	NEO extraversion		0.329	2.660	0.011
Nocebo hyperalgesia	*Model 1*	20.3			
	ASI		0.451	3.349	0.002
	*Model 2*	33.3			
	ASI		0.498	3.966	<0.001
	NEO openess		−0.364	−2.897	0.006
	*Model 3*	42.9			
	ASI		0.493	4.197	<0.001
	NEO openess		−0.472	−3.796	<0.001
	NEO extraversion		0.329	2.660	0.011
	*Model 4*	49.1			
	ASI		0.448	3.919	<0.001
	NEO openess		−0.413	−3.388	0.002
	NEO extraversion		0.387	3.197	0.003
	BDI		0.267	2.218	0.032

*ASI, Anxiety Sensitivity Index; BDI, Beck Depression Inventory; NEO, Neuroticism-Extraversion-Openness Inventory. Only significant values are shown. Excluded variables (not significant for the model): STAI, State and Trait Anxiety Inventory; BAI, Beck Anxiety Inventory; FOP. Fear of Pain; MASQ, Mood and Anxiety Symptom Questionnaire; PCS, Pain Catastrophizing Scale; PSWQ, Penn State Worry Questionnaire; PANAS, Positive and Negative Affective Schedule*.

Finally, we calculated mediation analyses for exploring the relationship among personality factors, positive/negative expectations and placebo/nocebo responses. Interestingly, we found that expectations were significantly linked to placebo and nocebo effects (see Table 5). However, personality factors *per se* did not influence expectancies, and the indirect effect among the three variables was not significant. Due to the exploratory nature of this part of the study, we used a relative broad battery. Therefore, correlations among personality questionnaires are shown in Table 6.

**Table 5:** **Mediation analysis results**.

**Model**	**a path**	**b path**	**c' path**	**Indirect effect**
BAI (X)	*p* = 0.094	*p* = 0.018	*p* = 0.099	*p* = 0.184
Positive expectations (M)				
Placebo hypoalgesia (Y)				
FOP severe (X)	*p* = 0.656	*p* = 0.005	*p* = 0.005	*p* = 0.673
Positive expectations (M)				
Placebo hypoalgesia (Y)				
FO*P* medical (X)	*p* = 0.656	*p* = 0.011	*p* = 0.012	*p* = 0.309
Positive expectations (M)				
Placebo hypoalgesia (Y)				
FOP total (X)	*p* = 0.217	*p* = 0.007	*p* = 0.012	*p* = 0.286
Positive expectations (M)				
Placebo hypoalgesia (Y)				
MISS physiol. (X)	*p* = 0.181	*p* = 0.010	*p* = 0.078	*p* = 0.321
Negative expectations (M)				
Nocebo hyperalgesia (Y)				
ASI (X)	*p* = 0.871	*p* < 0.001	*p* = 0.014	*p* = 0.879
Negative expectations (M)				
Nocebo hyperalgesia (Y)				
PCS rumination (X)	*p* = 0.493	*p* = 0.023	*p* = 0.006	*p* = 0.539
Negative expectations (M)				
Nocebo hyperalgesia (Y)				
PCS help. (X)	*p* = 0.322	*p* = 0.031	*p* = 0.022	*p* = 0.398
Negative expectations (M)				
Nocebo hyperalgesia (Y)				
PCS total (X)	*p* = 0.350	*p* = 0.027	*p* = 0.014	*p* = 0.419
Negative expectations (M)				
Nocebo hyperalgesia (Y)				

*BAI, Beck Anxiety Inventory; FOP, Fear Of Pain; MISS, Multidimensional Iowa Suggestibility Scale; ASI, Anxiety Sensitivity Index; PCS, Pain Catastrophizing Scale*.

**Table 6:** **Correlations among personality measurement tools**.

		**STAI post**	**STAI 2**	**ASI**	**BAI**	**BDI**	**PANAS**	**NEO**	**MASQ**	**TPQ**	**BIS/BAS**	**LotR**	**IMI**	**IRI**	**MISS**	**FOPtot**	**PCS**	**PSWQ**	**SS**
STAI_1 pre	Rho	0.690^**^	0.689^**^	0.277	0.153	0.336^*^	0.351^*^	0.029	0.607^**^	0.175	−0.054	−0.147	−0.041	0.057	0.072	0.213	0.214	0.170	0.020
	*p*-value	**0.000**	**0.000**	0.062	0.309	0.022	0.017	0.850	**0.000**	0.246	0.722	0.328	0.789	0.709	0.636	0.155	0.154	0.258	0.898
STAI_1 post	rho		0.582^**^	0.226	0.182	0.293^*^	0.482^**^	0.094	0.509^**^	0.310^*^	−0.046	−0.165	−0.098	−0.039	−0.032	−0.032	0.317^*^	0.213	−0.004
	*p*-value		**0.000**	0.130	0.225	0.048	**0.001**	0.533	**0.000**	0.036	0.760	0.272	0.518	0.796	0.832	0.830	0.032	0.154	0.980
STAI_2	rho			0.309^*^	0.376^*^	0.461^**^	0.456^**^	0.085	0.678^**^	0.240	0.199	−0.400^**^	−0.025	0.202	0.062	0.310^*^	0.192	0.392^**^	−0.115
	*p*-value			0.036	0.010	**0.001**	**0.001**	0.576	**0.000**	0.108	0.186	0.006	0.868	0.179	0.681	0.036	0.200	0.007	0.449
ASI	rho				0.433^**^	0.153	0.401^**^	0.076	0.474^**^	0.377^**^	−0.029	−0.129	0.053	0.355^*^	0.576^**^	0.181	0.561^**^	0.482^**^	−0.008
	*p*-value				**0.003**	0.310	0.006	0.615	**0.001**	0.010	0.848	0.394	0.725	0.015	**0.000**	0.229	**0.000**	**0.001**	0.958
BAI	Rho					0.242	0.269	0.157	0.446^**^	0.505^**^	0.017	−0.235	0.016	0.202	0.438^**^	0.401^**^	0.368^*^	0.376^**^	0.103
	*p*-value					0.104	0.070	0.297	**0.002**	**0.000**	0.912	0.115	0.915	0.177	**0.002**	0.006	0.012	0.010	0.496
BDI	rho						0.362^*^	−0.161	0.449^**^	0.112	0.129	−0.254	−0.201	−0.130	0.101	0.227	0.211	0.177	−0.118
	*p*-value						0.013	0.286	**0.002**	0.460	0.392	0.088	0.180	0.389	0.506	0.130	0.159	0.238	0.434
PANAS	Rho							0.668^**^	0.668^**^	0.303^*^	0.082	−0.460^**^	−0.285	−0.050	0.001	0.244	0.249	0.461^**^	−0.029
	*p*-value							**0.000**	**0.000**	0.041	0.588	**0.001**	0.055	0.740	0.994	0.103	0.096	**0.001**	0.849
NEO	Rho								−0.132	0.449^**^	−0.348^*^	0.475^**^	0.594^**^	0.602^**^	0.289	−0.058	−0.001	0.056	0.044
	*p*-value								0.381	**0.002**	0.018	**0.001**	**0.000**	**0.000**	0.052	0.700	0.994	0.711	0.774
MASQ	Rho									0.111	0.335^*^	−0.409^**^	−0.174	0.130	0.111	0.381^**^	0.276	0.381^**^	−0.155
	*p*-value									0.463	0.023	**0.005**	0.249	0.390	0.463	0.009	0.063	0.009	0.303
TPQ	Rho										−0.367^*^	0.020	0.236	0.327^*^	0.427^**^	0.147	0.312^*^	0.429^**^	0.119
	*p*-value										0.012	0.894	0.115	0.026	**0.003**	0.328	0.035	**0.003**	0.431
BISBAS	Rho											−0.366^*^	−0.122	−0.152	−0.294^*^	0.055	−0.069	−0.140	−0.472^**^
	*p*-value											0.012	0.420	0.312	0.047	0.714	0.649	0.355	**0.001**
LotR	Rho												0.521^**^	0.132	0.104	−0.195	0.040	−0.101	−0.034
	*p*-value												**0.000**	0.383	0.492	0.195	0.789	0.503	0.824
IMI	Rho													0.428^**^	0.344^*^	0.016	0.127	−0.008	−0.128
	*p*-value													**0.003**	0.019	0.918	0.401	0.960	0.395
IRI	Rho														0.494^**^	0.179	0.133	0.201	−0.180
	*p*-value														**0.000**	0.233	0.378	0.181	0.231
MISS	Rho															0.357^*^	0.450^**^	0.219	0.089
	*p*-value															0.015	**0.002**	0.143	0.554
FOP	Rho																0.113	0.399^**^	−0.007
	*p*-value																0.456	0.006	0.966
PCS_Tot	Rho																	0.305^*^	0.125
	*p*-value																	0.039	0.407
PSWQ	Rho																		0.071
	*p*-value																		0.639

*STAI 1-2, State and Trait Anxiety Inventory; ASI, Anxiety Sensitivity Index; BAI, Beck Anxiety Inventory; BDI, Beck Depression Inventory; PANAS, Positive and Negative Affective Schedule; NEO, Neuroticism-Extraversion-Openness Inventory; MASQ, Mood and Anxiety Symptom Questionnaire; TPQ, Tridimensional Personality Questionnaire; BIS/BAS, Behavioral Inhibition and Behavioral Activation Scale; LotR, Life-Orientation Test-Revisited; IMI, Intrinsic Motivation Inventory; IRI, Interpersonal Reactivity Index; MISS, Multidimensional Iowa Suggestibility Scale; FOP, Fear of Pain; PCS, Pain Catastrophizing Scale; PSWQ, Penn State Worry Questionnaire; SS, Sensation Seeking. Significant results are indicated in bold*.

* *p < 0.05*;

** *p < 0.010*.

## Discussion

In this study, we investigated the influence of expectations and hypothesized psychological factors on placebo and nocebo effects elicited by a well-established model of conditioning and heat thermal painful stimulation. Placebo hypoalgesic responses were negatively correlated with severity of anxiety and fear of pain (e.g., medical fear, severe, and total fear). On the contrary, nocebo hyperalgesic responses were positively correlated with anxiety sensitivity, suggestibility and catastrophizing (trend only). Moreover, a stepwise regression modeling showed that aggregate scores of Motivation (value/utility and pressure/tense subscales) and suggestibility (physiological reactivity and persuadability subscales) accounted for the 51% of the variance in the placebo responses. By contrast, the aggregation of anxiety, openness, extraversion and depression accounted for the 49.1% of the variance in the nocebo responses. Importantly, expectations were highly correlated with placebo and nocebo effects and psychological factors did not influence level of expectations towards reduction or increase of pain.

Consistently with previous studies (Colloca and Benedetti, 2006, 2009; Colloca et al., 2008, 2010; Lui et al., 2010), we found that visual cues associated with prior experiences of low and high pain elicit strong placebo and nocebo effects with a distribution raging from no responses to low modulation of pain, to medium and high reductions and increases (Figure 3). Studies on placebo hypoalgesia and nocebo hyperalgesia have shown a substantial inter-individual variability and distinct personality factors have been associated with placebo and nocebo effects (Colloca and Grillon, 2014; Colagiuri et al., 2015). There is evidence that some personality factors such as anxiety (Staats et al., 2001; Ober et al., 2012), fear of pain (Lyby et al., 2010) and neuroticism (Peciña et al., 2013), are associated with reduced placebo analgesia. We confirmed and expanded some of these findings. In our study, severity of anxiety as well as fear of pain (e.g., medical, sever, and total fear) were linked to reduced placebo responsiveness to pain. Severity of anxiety including symptoms of depression, feelings of hopelessness and irritability, guiltiness or feelings of being punished, as well as physical symptoms such as fatigue, correlated negatively with placebo responses with higher severity of anxiety linked to lower reduction of pain induced by positive expectations. High levels of fear of pain referring to the dispositional tendency to have negative emotions toward pain and pain anticipation have been also associated with placebo- and nocebo-induced pain modulation (Lyby et al., 2010; Aslaksen and Lyby, 2015). We found that fear of medical pain in particular correlates with low placebo hypoalgesic responses and this is consistent with the parallel enhancement of nocebo induced by fear of pain and other medical procedures (Aslaksen and Lyby, 2015).

When we looked at the nocebo effect—the negative counterpart of the placebo phenomenon (Petrovic, 2008)—we found a positive correlation with anxiety sensitivity, physiological suggestibility and catastrophizing. Anxiety sensitivity refers to behaviors or sensations associated with the experience of anxiety that elicit misinterpretations of bodily sensations such as the experience of a no harmful stimulus causing intense pain (Mehta et al., 2016). Suggestibility is a trait-like characteristic creating distinct behaviors that facilitate responsiveness to plausible information as well as inclinations to accept and act on others' suggestions in regards to the body (e.g., physical suggestibility), and has been linked to placebo effects (Lund et al., 2015) and nocebo effects (Corsi et al., 2016). Catastrophizing, a maladaptive cognitive process that is potentially heritable and has been reported to predict severity of clinical pain (Flor and Turk, 1988; Severeijns et al., 2001; Goubert et al., 2004; Kudel et al., 2005; Trost et al., 2015), has been recently explored and shown to be relevant for nocebo effects (Vogtle et al., 2013).

Personality is a continuum of factors and thus highlights the importance of considering distinct factors together. Therefore, based on the literature we took into consideration two sets of psychological factors related to placebo and nocebo responsiveness and used a multilevel modeling approach in which hierarchies and residual components at each level within a hierarchy are computed. Such an approach indicated that an aggregate score for motivation (value/utility and pressure/thanks subscales) and suggestibility (physiological reactivity and persuadability subscales) accounted for the 51% of the variance in the placebo hypolagesic responses whilst anxiety severity, NEO-openness-extraversion and depression considered together accounted for the 49.1% of the variance of nocebo responses suggesting that it helps evaluate the psychological factors comprehensively. Another important result from this study was that positive expectations were significantly correlated with placebo responses and negative expectations were significantly correlated with nocebo responses. Although one may argue that asking on a trial-by-trial about expectancy of the upcoming pain may have generated a sort of self-prophecy (e.g., You get what you expect, you get what you ask for), it remains an interesting finding that could be important to keep in mind every time we measure pain in real-world settings. Therefore, an obvious question was whether personality factors impact the formation of expectations of pain reduction and increase. In this study, mediation analyses indicated that personality factors (e.g., being worried, being fearful) had no direct effect on the level of expectation related to pain changes (e.g., reductions and increases). Future large scale studies deserve to be performed in pain patient and healthy populations to better understand the connection among psychological factors, expectancies, placebo and nocebo effects.

The inclusion of an extensive battery of questionnaires related to personality factors allowed us to reveal that expectations may predict placebo and nocebo effects independently of personality factors making it a helpful tool for health care providers.

Several studies have emphasized the need for exploring the impact of personality factors as at least one of the possible ways to interpret and understand the large variability in placebo analgesic and nocebo hyperalgesic responses. To our knowledge, this is the first study that explores how distinct psychological factors can predict placebo hypolagesic responses and nocebo hyperalgesic responses, and the potential influence of personality factors in shaping positive and negative expectancies. Collectively, the complexity and variability in placebo- and nocebo-induced pain responses highlight a need to better understand the multidimensionality of pain and its modulation related to individual expectations and psychological factors. This approach provides advantages in interpreting how pain is felt and experienced.

## Author contributions

LC and NC conceived the study design. NC performed the experiments. NC and LC analyzed the data and drafted the manuscript. LC revised and finalized the manuscript.

## Funding

This research was partially supported by the University of Maryland, Baltimore (LC), the National Institute of Dental and Craniofacial Research (1R01DE025946-0; LC) and the Cooperint Internalization Program from University of Verona, Italy (NC).

### Conflict of interest statement

LC has received lecture honoraria (Georgetown University and Stanford University) and has acted as speaker or consultant for Grünenthal and Emmi Solution. NC has no conflicts of interest to be declared. The reviewer JG and the handling Editor declared their shared affiliation, and the handling Editor states that the process nevertheless met the standards of a fair and objective review.
